# The Patient- And Nutrition-Derived Outcome Risk Assessment Score (PANDORA): Development of a Simple Predictive Risk Score for 30-Day In-Hospital Mortality Based on Demographics, Clinical Observation, and Nutrition

**DOI:** 10.1371/journal.pone.0127316

**Published:** 2015-05-22

**Authors:** Michael Hiesmayr, Sophie Frantal, Karin Schindler, Michael Themessl-Huber, Mohamed Mouhieddine, Christian Schuh, Elisabeth Pernicka, Stéphane Schneider, Pierre Singer, Olle Ljunqvist, Claude Pichard, Alessandro Laviano, Sigrid Kosak, Peter Bauer

**Affiliations:** 1 Department of Anaesthesiology, General Intensive Care and Pain Control, Division Cardiac-, Thoracic-, Vascular Anaesthesia and Intensive Care, Medical University Vienna, Vienna, Austria; 2 Center for Medical Statistics, Informatics and Intelligent Systems, Section for Medical Statistics, Vienna, Austria; 3 Medical Clinic III, Division Endocrinology, Medical University Vienna, Vienna, Austria; 4 Nutritional Support Unit, Pôle Digestif, Hôpital de l’Archet, Nice, France; 5 General Intensive Care Department, Rabin Medical Center University Hospital, Beilinson Campus, Petah Tiqwa, Israel; 6 Department of Surgery, Faculty of Medicine and Health, Örebro University, Örebro, Sweden; 7 Clinical Nutrition, Geneva University Hospital, Geneva, Switzerland; 8 Department of Clinical Medicine, University La Sapienza, Rome, Italy

## Abstract

**Objective:**

To develop a simple scoring system to predict 30 day in-hospital mortality of in-patients excluding those from intensive care units based on easily obtainable demographic, disease and nutrition related patient data.

**Methods:**

Score development with general estimation equation methodology and model selection by P-value thresholding based on a cross-sectional sample of 52 risk indicators with 123 item classes collected with questionnaires and stored in an multilingual online database.

**Setting:**

Worldwide prospective cross-sectional cohort with 30 day in-hospital mortality from the nutritionDay 2006-2009 and an external validation sample from 2012.

**Results:**

We included 43894 patients from 2480 units in 32 countries. 1631(3.72%) patients died within 30 days in hospital. The **P**atient- **A**nd **N**utrition-**D**erived **O**utcome **R**isk **A**ssessment (PANDORA) score predicts 30-day hospital mortality based on 7 indicators with 31 item classes on a scale from 0 to 75 points. The indicators are age (0 to 17 points), nutrient intake on nutritionDay (0 to 12 points), mobility (0 to 11 points), fluid status (0 to 10 points), BMI (0 to 9 points), cancer (9 points) and main patient group (0 to 7 points). An appropriate model fit has been achieved. The area under the receiver operating characteristic curve for mortality prediction was 0.82 in the development sample and 0.79 in the external validation sample.

**Conclusions:**

The PANDORA score is a simple, robust scoring system for a general population of hospitalised patients to be used for risk stratification and benchmarking.

## Introduction

Hospitals are major providers of health care services and may consume up to 50% of the national health care budget. Variability in services provided, differences in outcome, and resource utilization are major concerns [1]. Prognostic information about patients is important to evaluate the effectiveness of services provided in the health care system [2] and to compare treatment approaches as well as hospitals [3,4]. Publicly disclosed mortality figures for individual hospitals may not offer a fair comparison between institutions because of the difficulties in adjusting for the case-mix [5–8]. Thus the estimates of severity of illness may not be comparable between hospitals and more so between countries. The usefulness of such public disclosure has been challenged because of poor association with quality [6], selection bias [5], sensitivity to coding practice [7,9], type of standardisation[10], and missing transparency of models [11].

The data source used in such benchmarking efforts may affect the result. Administrative data, e.g. accounting data [12] are easily available but accuracy depends on local coding practice[13] whereas case note extraction [14] and specific data collection is time and resource consuming. In addition administrative data are only available after discharge when International Classification of Diseases codes (ICD-10) [15] have been assigned to patients. Case-mix adjustment is either done by regression analysis within diagnostic groups [3], by using the Charlson Comorbidity Index [12] or adding Current Procedural Terminology codes in the surgical field [16]. When applying such scores to external samples, estimation of mortality may be poor and often necessitates recalibration, e.g. to regions or countries [17]. Some of the models for case-mix adjustment for general populations of hospitalised patients are not disclosed by the commercial providers working with administrative[18] or case note[14] data., making it a “black box” [11]. This “black box” and inclusion of 28 to 95% of patients admitted during the observation period [19] obscures understanding of institutional strength and weakness.

Predictive models have been extensively used in acute care such as medical emergency admissions[20], intensive care admissions [21,22] or specific acute conditions such as stroke, acute coronary syndrome [23], heart failure [24] or pneumonia. In addition to predict death, risk models are widely used to predict complications such as bleeding or nosocomial infections in specific situations. Prognostic models for acute care situations always include either several vital signs typically obtained from monitors, electrocardiograms or imaging information, biomarker or procedural characteristics. This information is missing in a large proportion of patients even in medical emergency departments [25]. There is no prognostic model quantifying the risk in the population of hospitalised patients as a whole—excluding ICU-patients—by a single scoring system.

The aim of this study was to develop a prognostic model and to derive a single prognostic score for 30-day in—hospital mortality of in-patients excluding ICU-patients that are already covered by specific systems [21,22,26–29]. The elements of such a predictive score should be easily obtainable from the patient’s history, interview, and direct observation at any time during hospitalisation, even before a definitive diagnosis is known. To that end we used the data from the nutritionDay survey which uses cross-sectional samples from different years and countries with a focus on robustly available information on patient characteristics and nutrition.

## Methods

### Study design

The PANDORA scoring system to predict 30-day in-hospital mortality of adult hospitalised patients was developed in a three-step process based on variable selection, parameter estimation, and score validation that followed the PROGRESS suggestions [2].

### Population

**Fig 1:**
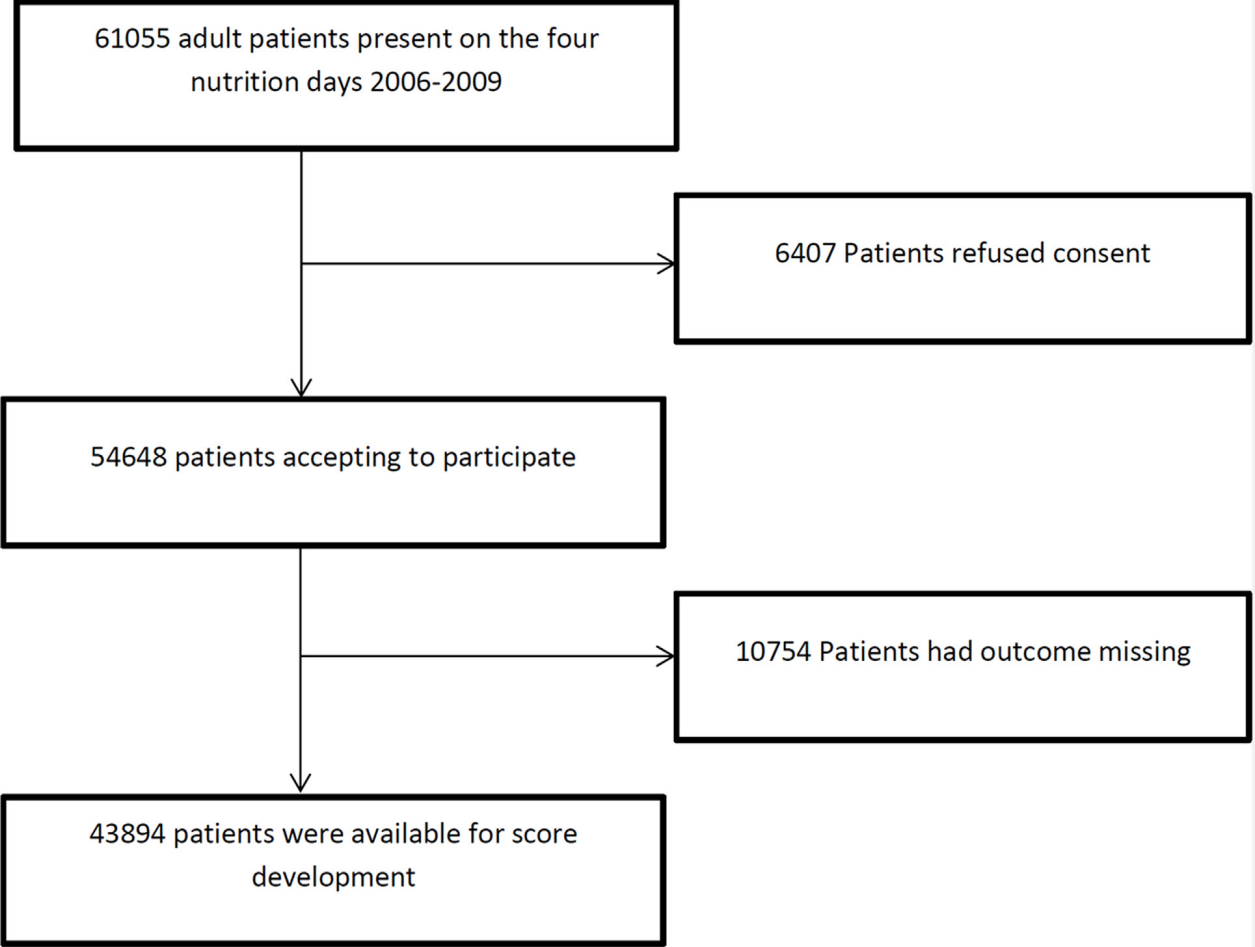
Flowchart of the selection process for inclusion in the PANDORA score development sample.

For development of the score, we used the data from four cross-sectional data collections, the “nutritionDay” 2006–2009 [30] (www.nutritionday.org) surveys held on 19.01.2006, 25.01.2007, 31.01.2008, and 29.01.2009. The nutritionDay study is a survey for hospital wards (ICU patients were not included) with voluntary participation and registration via an open web based application. In total, 61055 patients were present in the participating wards during the four surveys (Fig 1). The nutritionDay survey collected simple demographic data, including patients’ preadmission nutrition history, organs affected, comorbidities, mobility, simple indicators of intensity of care and actual nutrition intake. The main outcome parameter prospectively collected was patient’s status (death, transferred to another hospital, transferred to long-term care, discharged home) at discharge until 30 days after nutritionDay. The relationship between nutrition related risk factors and death was one of the main goals of the study[30].

No specific measurement or specialised knowledge was necessary for data acquisition. One to two workdays were necessary to obtain and disseminate information about the survey, download and distribute questionnaires to patients, collect medical information, and enter data in the multilingual online database for 20–30 patients per ward. The only incentive to participate was to receive a summary report for the individual unit in comparison with similar wards. The nutritionDay project was approved by the Ethical Committee of the Medical University Vienna (EC number, 407/2005) and by local ethical committees (S1 Table) if required by national rules. The database is open upon request along the “Guidelines for Project submission” at http://www.nutritionday.org/en/researchers-scientists/researchers-scientists/publish-nday-results/index.html for interested parties that submit a research protocol to office@nutritionday.org.

For the present study we included all adult patients for whom date and status at discharge were available. Hence, 43894 patients are part of the score development data set (Fig 1). The survey in 2012 had a sufficient overlap of variables to be used for validation of the score with an independent sample. All available patient data were used to develop the PANDORA score. Variables not collected in one (or more) of the five years are not used in the score-building process. Overall, 3.3% of extractable data were missing and were therefore imputed. Multivariate imputation of missing data was done by using predictive mean matching using the aregImpute and transcan algorithm in R [31].

To avoid patient selection, we based the score on the imputed data set, because hospital mortality in patients with missing values was noticeably higher than that in complete cases (5.2% vs. 2.6%). Nevertheless, a sensitivity analysis for score development based on the non-missing fraction of the non-imputed data was done.

### Variable selection

First, to achieve a simple coding system in the final score, we divided most metric variables such as age, length of hospital stay, and BMI into categories. The cut-points were chosen according to existing proposals (World Health Organization for BMI, 10-years age groups) or simplicity (e.g. duration of stay in hospital more or less than 2 weeks). The categorical variable “food intake” was highly correlated between breakfast, lunch, and dinner. To keep the score simple, we included only the main meal for score development. Hence, 52 variables with 123 item classes were used for score development (S2 Table).

In a second step, to achieve a reasonable number of items assuring convergence in the following multivariate models, a pre-screening by univariate generalized estimating equations for “death in hospital within 30 days” were performed. Clinical wards were considered as repeated factor to account for the clustering of patients. Only variables which show an association with mortality with a p-value < 0.2 were included in multivariate modelling. Based on this crude pre-selection a multivariate generalized estimating equations approach (clinical wards as repeated factor) was applied for model building (SAS Procedure genmod). A smaller than usual local significance level of 0.01 was applied to select the variables for the final model because it is known that model selection by multiple p-value thresholding may serve as a consistent model selection procedure if the threshold for the individual p-values is decreased for increasing sample sizes[32]. Since consistency over time is an important issue we added an additional criterion for variable selection to derive a stable small prognostic model. The previously described selection process was applied to the subsamples of the four years 2006–2009. To account for the smaller sample sizes in the annual subsamples the threshold for including a variable into the model has been increased to 0.05. Variables were only included in the small prognostic model if selected in all four annual subsamples. To investigate the impact of such a very conservative selection criterion, we repeated model selection without the stability criterion over calendar time and compared the performance between the resulting extended model and the basic model.

### Parameter estimation / Construction of the Score

The parameters for the final score were then estimated by another multivariate generalized estimating equation model including only the independent variables selected in the step before. The final coefficients for the score were determined by multiplying by 10, rounding to the next integer toward the origin, and thus providing easily usable numbers and potentially improving prediction [33].

### Validation of the score

We assessed the performance of the score by using standard measures as the maximum R2 (Max R^2^), aROC, Brier score, and Hosmer-Lemeshow goodness-of-fit C-statistic[34].

Corresponding score estimates from 500 bootstrap samples randomly drawn (with replacement) from the development sample were used to calculate Harrell’s optimism. The whole model building process was repeated for each bootstrap sample. Every constructed model was validated in the bootstrap sample and the original sample. The mean difference serves as an estimator of the optimism [35].

The performance of the score was also investigated in an external validation sample from nutritionDay 2012 (N = 12928), which was available after score development from the data of 2006–2009.

### Sensitivity analyses

As a sensitivity analysis we repeated variable selection and score estimation based on a data set including only patients with complete data. In a second sensitivity analysis all units with less than 95 percent of outcome data were excluded and a separate imputation of missing data was performed in this subsample. Furthermore we looked how the score derived from the imputed data set performs in the patients without missing values, either in the development or in the validation sample.

### Programs

The statistical analyses were done with the software programs SAS 9.4 (SAS statistical software, SAS Institute, Cary, NC) and R 3.1.1 (R_Development_Core_Team 2008)[36].

## Results

The development sample (2006–2009) included 43894 patients from 2480 wards participating in an individual year; 1631 patients (3.72%) died in the hospital within 30 days after nutritionDay. The median (lower quartile; upper quartile) of the observed time between hospital admission and death was 24 (14; 36). The validation sample from the year 2012 included 12928 patients. In Table 1 the distributions of the variables selected in the PANDORA or in the extended score out of the 52 candidates are described for both samples together with the geographical region and comorbidities.

**Table 1:** Distribution of variables for the development sample 2006–2009 and the validation sample 2012.

Variable	Development Sample NutritionDay 06–09 N = 43894		Validation Sample NutritionDay 12 N = 12928	
**Death**	1631	3.7%	535	4.1%
**Sex (Female)**	21976	50.1%	6320	48.9%
**Age (years)**				
< 40	4831	11.0%	1656	12.8%
40–50	4250	9.7%	1296	10.0%
50–60	6504	14.8%	2028	15.7%
60–70	8561	19.5%	2524	19.5%
70–80	9580	21.8%	2669	20.7%
80–90	8129	18.5%	2212	17.1%
> 90	2039	4.7%	543	4.2%
**BMI (kg.cm** ^**-2**^ **)**				
< 18.5	3136	7.1%	1055	8.2%
18.5–25	19666	44.8%	5515	42.6%
25–30	13551	30.9%	3771	29.2%
30–35	5203	11.9%	1643	12.7%
35–40	1532	3.5%	589	4.6%
> 40	806	1.8%	355	2.7%
**Can you walk?**				
Without assistance	29440	67.1%	7716	59.7%
Only with assistance	9283	21.1%	3557	27.5%
I stay in bed	5171	11.8%	1655	12.8%
**Nutrition intake last week**				
normal	22275	50.8%	6372	49.3%
< normal	10128	23.1%	2884	22.3%
< half of normal	6249	14.2%	1901	14.7%
< a quarter to nothing	5242	11.9%	1771	13.7%
**What did you eat today?**				
All	16437	37.4%	5192	40.2%
Half	13576	30.9%	3377	26.1%
Quarter	5472	12.5%	1981	15.3%
Nothing, allowed	4594	10.5%	1238	9.6%
Nothing, not allowed	3815	8.7%	1140	8.8%
**Receiving Additional Nutrition**	9059	20.6%	2955	22.9%
**LOS > 14 days**	11011	25.1%	3122	24.2%
**Duration since operation**				
No operation	29911	68.1%	8411	65.0%
0–3 days	7677	17.5%	2699	20.9%
4–7 days	2086	4.8%	515	4.0%
> 7 days	4220	9.6%	1303	10.1%
**Diseased organs** *				
Lung	5404	12.3%	1906	14.7%
Liver	3073	7.0%	614	4.7%
Gastrointestinal tract	10119	23.1%	2519	19.5%
Skeleton/Bone/Muscle	6945	15.8%	2042	15.8%
Cancer	7530	17.2%	2273	17.6%
Other	34486	78.6%	9811	75.9%
**Comorbidity** *				
Cardiac Insufficiency	4679	10.7%	1419	11.0%
Diabetes I/II	7155	16.3%	2484	19.2%
Stroke	2070	4.7%	665	5.1%
COPD	2388	5.4%	830	6.4%
Myocardial infarction	1744	4.0%	414	3.2%
Other	15150	34.5%	5791	44.8%
**Fluid Status**				
Dehydrated	3942	9.0%	1116	8.6%
Overhydrated	4790	10.9%	1577	12.2%
Normal	35162	80.1%	10235	79.2%
**Speciality of Ward**				
Internal	13919	31.7%	4504	34.8%
Surgery	21653	49.3%	6285	48.6%
Geriatrics	2781	6.3%	1030	8.0%
Neurology	1514	3.5%	156	1.2%
Others	4027	9.2%	953	7.4%
**Regions**				
Central Europe	19867	45.3%	1237	9.6%
Northern Europe	3283	7.5%	620	4.8%
Southern Europe	7998	18.2%	2422	18.7%
Western Europe	6601	15.0%	2441	18.9%
Eastern Europe	2567	5.8%	106	0.8%
America	582	1.3%	3318	25.7%
Eastern Mediterranean	680	1.6%	895	6.9%
Western Pacific/SE Asia	2316	5.3%	1889	14.6%

* multiple answers possible

### The PANDORA score

The resulting PANDORA score (Table 2) was based on 7 indicators with 31 item classes. The score is built by summing the individual item values (Table 2) and has a theoretical minimum of 0 and a maximum of 75. The minimum observed value was 0, and the maximum observed value was 71 with a mean of 26.4 ± 11.2 (SD). Three indicators were provided by physicians and nurses, and four were provided by the patients. All selected variables contributed substantially to the PANDORA score.

**Table 2:** PANDORA additive score values to predict 30 day hospital mortality*.

Variable	Groups	Score
Age	<40	0
	40–50	6
	50–60	8
	60–70	10
	70–80	11
	80–90	14
	> = 90	17
Body Mass Index (BMI)	<18.5	9
	18.5–25	6
	25–30	2
	30–35	0
	35–40	0
	>40	3
Can you walk?	Walk without assistance	0
	Only with assistance	6
	I stay in bed	11
What did you eat today?	All	0
	Half	3
	Quarter	9
	Nothing, Allowed	12
	Nothing, Not allowed	7
Main patient group admitted	Internal	7
	Surgery	0
	Geriatrics	5
	Neurology	3
	Others	6
Diseased Organ	Cancer	9
Fluid status	Dehydrated	7
	Normal	0
	Overload	10
**PANDORA score**	**sum**	

* The relationship between the PANDORA score and hospital mortality within 30 days is given by the equation: logit = -6.72 + 0.1058 x PANDORA score. The probability of death is given by the equation: Probability of death = e^logit^ / (1+e^logit^).

The relationship between the PANDORA score and hospital mortality within 30 days is given by the equation: logit of death = -6.72 + 0.1058 x PANDORA score, and the probability of death is given by the equation: Probability of death = e^logit^ / (1+e^logit^).

**Fig 2:**
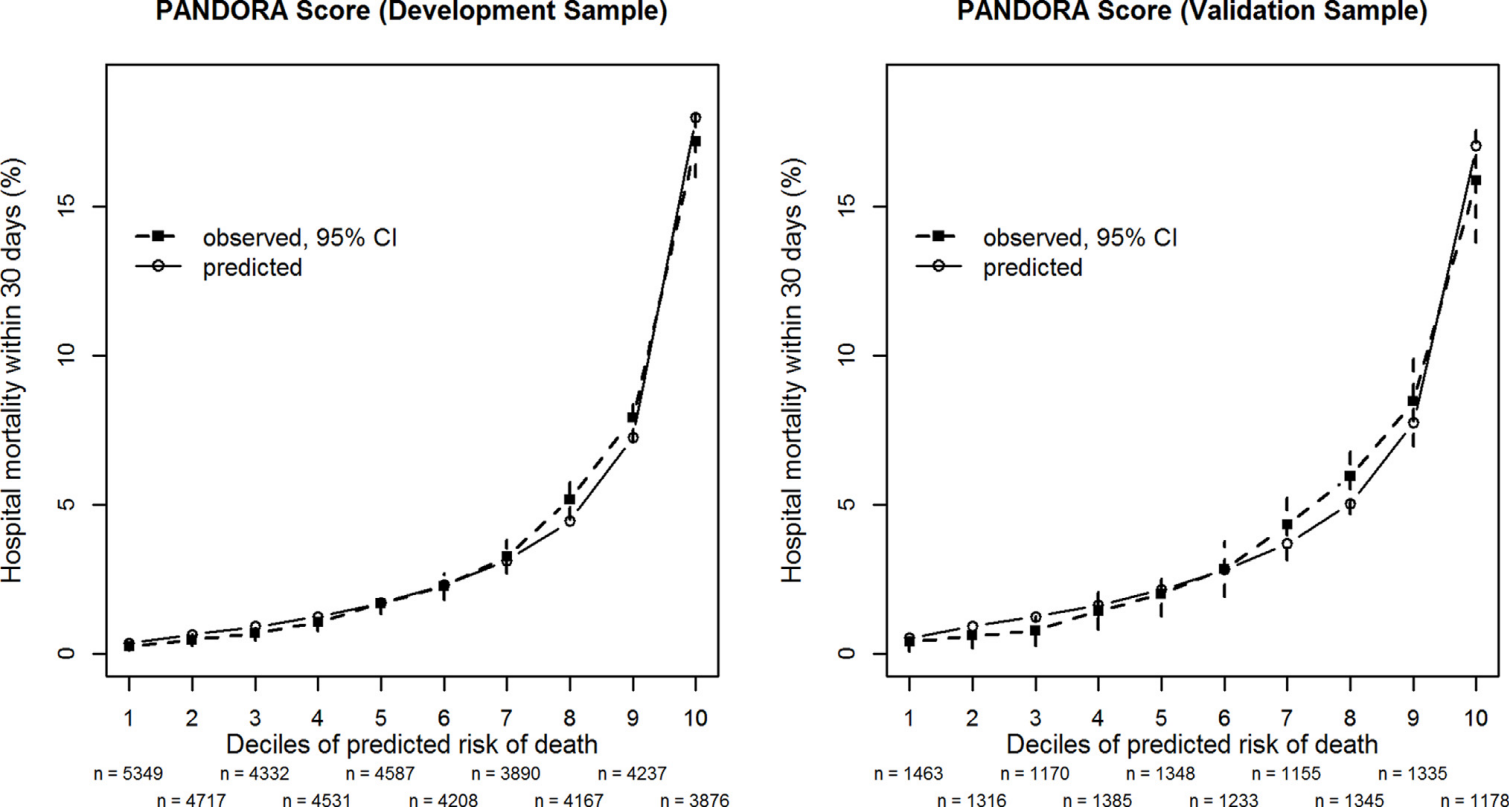
Observed and predicted hospital mortality by the PANDORA score. Patients are grouped by decile-classes of predicted in-hospital mortality within 30 days after the cross-sectional survey derived from the PANDORA score for the development sample (left panel) from the years 2006–2009 (n = 43894) and the external validation sample (right panel) from the year 2012 (n = 12928). The numbers of patients in each decile (n) are given below the x-axis. Closed symbols (■) show observed mortality with 95% confidence intervals (CI) whereas open symbols (⦿) show predicted mortality. The PANDORA score has 7 indicator variables (Table 2).

The average discriminatory capability of the model, as measured by aROC, was 0.817 in the development sample. Max-rescaled R^2^ was 0.187 and the Brier score was 0.033. For all score deciles there was good agreement between observed and predicted mortality in the development sample and the external validation sample (Fig 2). The Hosmer-Lemeshow test for predicted mortality in deciles of expected risk indicated a lack of fit (C-statistic, chi-square = 18.38, df = 8, p = 0.02). However, due to the large sample size, even relatively small deviations between actual and estimated mortality (Fig 2) would result in small p-values. The optimism-corrected results were similar, the aROC was then 0.815, the max-rescaled R² was 0.183 and the Brier score was almost the same as before.

Multi-collinearity was no problem at all. The correlations between predictors were surprisingly low to moderate and we had no problems with collinearity in the multivariate analysis with any of the 500 bootstrap samples. When adding the two-fold interactions among the seven variables none of them succeeded to contribute to the model in a consistent way over the years. None of the interactions resulted in a p-value lower than 0.1 in all four years simultaneously.

In the external validation sample 12928 patients were available. Surprisingly, the Hosmer-Lemeshow test (C-statistic, chi-square = 12.84, df = 8, p = 0.12) in the smaller external validation sample did not indicate any noticeable lack of fit (see also Fig 2). The area under the ROC was 0.787, Max-rescaled R² was 0.149 and the Brier score was 0.037.

When, in the sensitivity analysis, the same method was applied to construct the score based only on data of the 25307 patients without any missing value among the 52 candidate variables the value of the resulting aROC was 0.816. One remarkable difference is that BMI is not included in the score built from complete cases. Note that BMI was the variable in the PANDORA score that almost failed to pass the strong stability criterion of being selected in every year of the imputed development sample. In the separate selection process for the year 2007 of the sensitivity analysis the actual amount of nutrient eaten did not enter the model. Instead the amount eaten last week entered the model. Due to the existing correlation between these two variables we nevertheless included the actual amount eaten in the model, which has been selected for all the other three years. The contribution of mobility increased to 13 points, whereas the contribution of the correlated variable age decreased to a maximum of 13 points. It should be noted that applying the PANDORA score (Table 2) to the non-missing data sample show a good discriminatory performance in the development (n = 25307, aROC = 0.821) as well as in the validation sample (n = 5187, aROC = 0.824).

In the second sensitivity analysis excluding units with less than 95 percent outcome data (n = 35046) resulted in an aROC of 0.819 in the development sample. In the external validation sample (n = 11422) the value of the aROC was 0.776. Minor changes in the score were observed and again the BMI was not selected.

### The extended score

The extended score (S3 Table) constructed by leaving out the rigid criterion of including variables only if selected in all 4 calendar years of the development sample was based on 11 indicators with 51 item classes. The score now had a theoretical minimum of -5 and a maximum of 85. The minimum observed value was -3, and the maximum observed value was 72 with a mean of 25.7 ± 11.3(SD). Six indicators were provided by physicians and nurses, and five were provided by the patients, for the distribution of the variables in the development and validation sample (see Table 1). Note that the shared variables in the PANDORA and extended score showed very similar contribution in both scores (Table 2 and S3 Table).

The extension of the score slightly improved the performance as compared to the PANDORA score: the area under the ROC was 0.832, Max-rescaled R² was 0.206 and the Brier score was 0.033. Again, the Hosmer-Lemeshow test (C-statistic, chi-square = 25.80, df = 8, p = 0.001) indicated some lack of fit in the large development sample. In the external validation sample the value for the aROC was 0.796, Max-rescaled R² was 0.165 and the Brier score 0.037. It is worth to note that the Hosmer-Lemeshow test (C-statistic, chi-square = 12.08, df = 8, p = 0.15) again did not indicate any noticeable lack of fit in the smaller external validation sample (S1 Fig).

## Discussion

We developed a basic scoring system for hospitalised patients to predict in-hospital death within 30 days from data of the nutritionDay survey (Table 2). The strength of the resulting PANDORA score, besides the model fit, is its simplicity. The seven variables entering the score do not require specialist knowledge. The score allows quantifying what is usually considered as the clinical impression of a patient staying in hospital: age, mobility, food intake, hydration status or oedema. In addition the specialty of the ward where the patient is staying and the presence of cancer add to the risk of death. Furthermore, the score does not require any procedural or health care system associated variables.

The large international sample of hospitalised patients from the nutrition Day survey has several advantages for the development of a prognostic score. Harmonised data have been collected prospectively in many countries, thus preventing a strong effect of particular national healthcare policies. Compared with administrative data no bias associated with local coding practice was foreseen. The spectrum of patients available for analysis is large because 18 different medical specialties participated. The barriers for participation in the study were lowered by providing questionnaires in 25 national languages, requesting no fee, and protecting the ward’s anonymity.

We chose a rigid model selection procedure based on a very large sample with an additional criterion of stability of selection over individual calendar years: Predictors were included into the model only when they contributed in a consistent and statistically convincing way.

The performance of the score with an aROC of 0.82 in the development sample and 0.79 in the external validation is not noticeably inferior to that in the specific scoring systems commonly used in intensive care[21,22,26–29]. We are convinced that this performance is satisfactory when considering the simplicity of our predictor variables as compared to the large set of physiological variables routinely available for prediction in ICU-patients. Overall there was an appropriate agreement between predicted an observed mortality in the development and particularly in the external validation sample. Note, that the external validation sample, although collected “internally” in the long term nutritionDay survey, had a quite different geographical distribution than the development sample (Table 1).

Not surprisingly the fit tends to be poorer for very large predicted mortalities, which is only covered by a limited number of patients. Consequently, the score is somehow dominated by patients with moderate to low risk. It is worth noting that for high predicted mortalities an increase of one point in the PANDORA score is associated with a much larger increase in predicted mortality than a one point increase for low predicted mortality. This also aggravates the problems of achieving a perfect fit in high risk patients. One of the problems of such scores is stability over time. By our way of constructing the PANDORA score we only included variables that significantly contributed to the models developed separately in each of the four calendar years 2006 to 2009. A further indication for stability over time is the good fit in the delayed external validation sample from the year 2012.

We also calculated an extended score by dropping the rigid condition of consistency in variable selection over the individual calendar years. This score is based on 11 variables. The new variables entered in the extended score are duration since hospital admission, having well eaten during last week, receiving additional nutrition, sex and affected organs. Only a small improvement of the performance with an aROC of 0.83 in the development sample and 0.80 in the external validation sample has been observed. This indicates that the PANDORA score is performing sufficiently as compared to the “extended” score which requires the collection of five additional variables covering also some procedural and health care related aspects without achieving any noticeable improvement in prediction and fit.

One of the further strengths of the scores developed in the context of the nutritionDay survey is the possibility of regular updates resulting from recent data. It is to be expected that the simple variables entering the scores will not be changed in future surveys.

Another problem to be considered is whether the score would perform adequately when it is applied routinely outside the nutritionDay survey. We cannot supply any data on that issue but conjecture that due to the simplicity of the variables particularly in the PANDORA score no noticeable bias from external use should arise.

There are some weaknesses connected with the score. In the nutritionDay project the focus was on simple items typically included in patient’s medical history, items associated with outcome and items included in existing screening scores for malnutrition[37,38]. An additional criterion was that items could be obtained from patients and caregivers without extensive training or expert knowledge. Therefore, no physiological or laboratory variables of the patients were available to be used for risk analysis. We believe that this weakness turns out as strength of the score, which solely is based on simple and easily accessible variables, and can also be used in countries with insufficient health-system capacity. However, this specific data structure inevitably does not allow the calculation of other scores such as the Charlson Comorbidity Index [12] from our data to allow comparison with the PANDORA score.

It is important to note, that cross-sectional sampling is biased with regard to length of stay of patients in hospital: patients with a longer stay are more likely to be included in the sample. Therefore we included a variable “time since hospital admission” into the candidate variables for the score which did not show a convincing contribution to the PANDORA score.

We cannot exclude the possibility that some units deliberately included patients with nutrition problems because of the weight given to nutrition in the survey.

A further weakness of the study is the large number of patients with missing outcome data. However, a sensitivity analysis including only wards with documented outcome in more than 95 percent of their patients did not noticeably change the prediction.

We cannot estimate the effect of imputing missing values on score development, but we performed a sensitivity analysis with only patients who had complete values, and that analysis yielded a similar selection, weight of items and performance. Moreover the PANDORA score shows good performance when applied only to the subsample of complete cases in the development as well as in the validation sample.

A further concern is that only short term outcome was recorded in this study. To this end it is important to note, that due to the cross-sectional sampling and outcome recording 30 days after the sampling day the time from hospital admission until death of in-patients varied between 1 and 202 days. A proportion of 35% of death has been observed after a hospital stay of more than 30 days. Thus, the score also includes deaths after 30 days in hospital. Since also widely used ICU-scores[21,22,26–29] are restricted to 30 day hospital mortality, we believe that the simple, easily applicable PANDORA score for non-ICU patients in hospitals may be considered as a valuable instrument for risk stratification and benchmarking. We commit, that studying the performance of the score in a variety of treatment venues will be necessary to evaluate its utility and may lead to a customization of the score to different environments.

In the nutritionDay project transfer to long-term care was another possible outcome. This type of outcome was predicted by a different pattern of variables not including any nutrition related factors. Age was the dominant factor and “dependency on help” and “brain and nerves being affected” turned out as additional predictors. Therefore we did not consider to analyse a composite outcome criterion.

To our surprise, we found that functional status [39], such as mobility and nutrition[40] were of major importance. Reduced mobility has recently also been included in the Simple Clinical Score for medical emergency patients in addition to vital signs [25]. Both poor nutrition and reduced mobility could be consequences of the disease process, but they may have health-related effects of their own. It is probable that nutrition-related factors are surrogates for unobserved patient characteristics that directly cause death.

The seven items selected for the PANDORA score cannot be compared in any meaningful way to the 500 key clinical findings extracted from medical records by the Medical Illness Severity Grouping System [41], in which 85 primary diagnoses accounted for 80% of deaths [18]. These findings have been supplanted by 56 primary diagnoses in the Dr Foster Intelligence database [3,7], or the 17 Charlson comorbidities derived from ICD-10 codes [42]. There is no need to recalculate the score in each diagnostic category, as is done in the Dr Foster Intelligence System [7], because major indicators related to medical specialty or organs affected are already included.

In summary, we propose a very simple, robust scoring system (PANDORA) with a surprisingly high performance in predicting in-hospital mortality with three strengths. First, our approach is based on the data available at any time during hospitalisation; second, little additional time is necessary because items are part of a narrative patient history; finally, the model is public, international, and independent of national coding conventions. We expect that the most important areas of application of the PANDORA score will be hospital quality control, benchmarking between institutions and risk stratification in clinical studies. Hospitals implementing a new electronic health care record system may include, the items for the score in their routine documentation, if not contained anyway.

## Supporting Information

S1 Fig Comparison of the performance of the PANDORA score and the extended score. Observed mortality versus predicted mortality. The data points represent observed mortalities (%) with 95% confidence intervals in the ten deciles of predicted mortality (%). The upper panels represents the development sample from the years 2006–2009 and the lower panels the external validation sample from 2012. The left panels’ show the result for the PANDORA score with 7 indicator variables (Table 2), the right panels those for the extended score with 11 indicator variables (S3 Table).
(TIF)

S1 Table List of countries with approving ethical committees. (DOCX)

S2 Table Indicator variables and item classes used for score development. Reference categories are marked in bold characters.
(DOCX)

S3 Table Extended additive score values to predict 30 day in-hospital mortality. The relationship between the extended score and in-hospital mortality within 30 days after the day of the cross-sectional survey is given by the equation: logit = -6.79 + 0.1091 x extended score. The probability of death is given by the equation: Probability of death = e^logit^ / (1+e^logit^).
(DOCX)
