# Correction: The Patient- And Nutrition-Derived Outcome Risk Assessment Score (PANDORA): Development of a Simple Predictive Risk Score for 30-Day In-Hospital Mortality Based on Demographics, Clinical Observation, and Nutrition

**DOI:** 10.1371/journal.pone.0158023

**Published:** 2016-06-16

**Authors:** The PLOS ONE Staff

The tenth author’s name is spelled incorrectly. The correct name is: Olle Ljungqvist.
