# Supplementary figures and images for: The Patient- And Nutrition-Derived Outcome Risk Assessment Score (PANDORA): Development of a Simple Predictive Risk Score for 30-Day In-Hospital Mortality Based on Demographics, Clinical Observation, and Nutrition
Source: PLoS One. 2015 May 22;10(5):e0127316. doi: 10.1371/journal.pone.0127316 (PMC4441510; doi:10.1371/journal.pone.0127316)

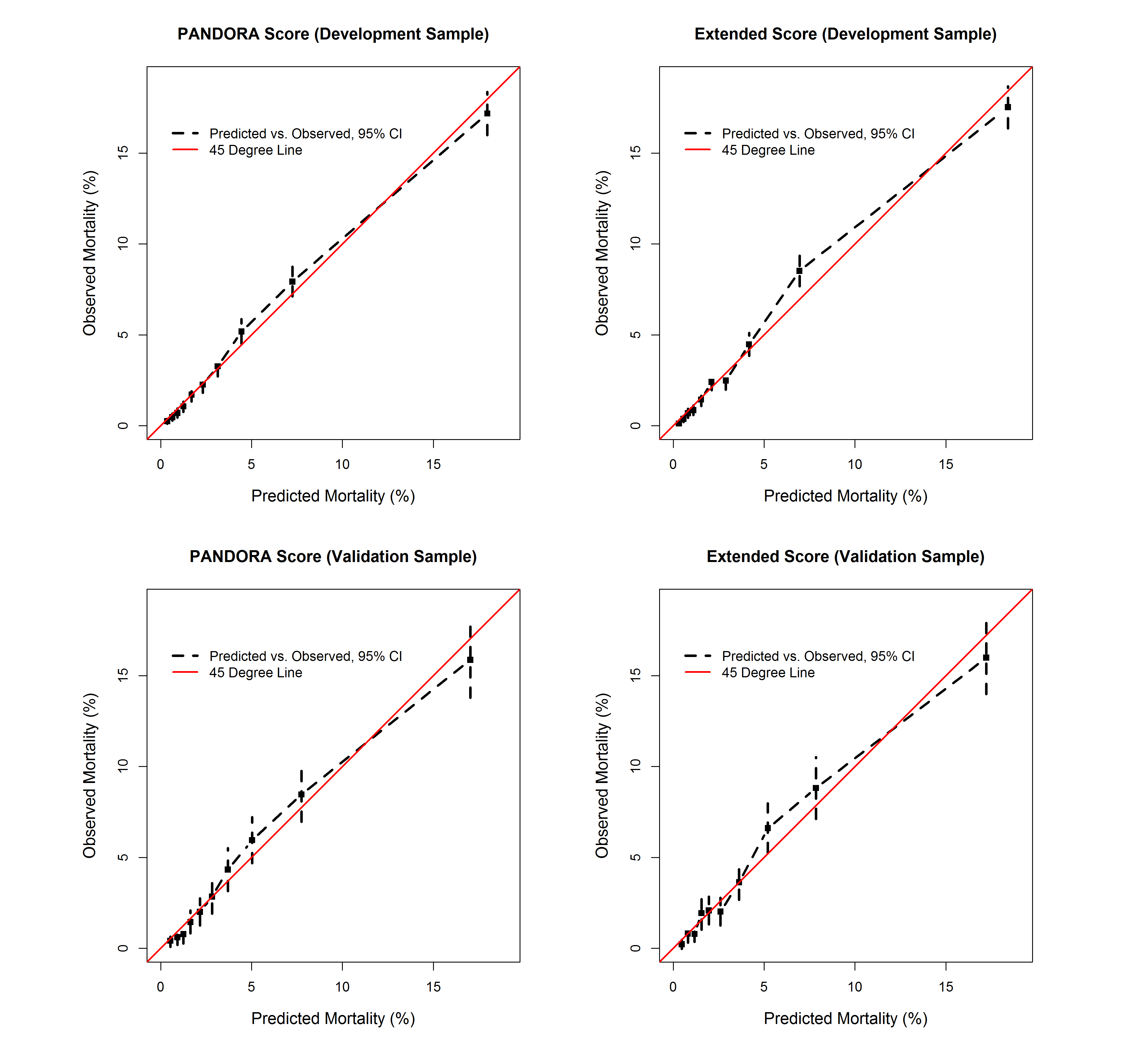

Supplement: S1 Fig — Observed mortality versus predicted mortality. The data points represent observed mortalities (%) with 95% confidence intervals in the ten deciles of predicted mortality (%). The upper panels represents the development sample from the years 2006–2009 and the lower panels the external validation sample from 2012. The left panels’ show the result for the PANDORA score with 7 indicator variables (Table 2), the right panels those for the extended score with 11 indicator variables (S3 Table). (TIF) [file pone.0127316.s001.tif]
