# Supplementary material for: The Patient- And Nutrition-Derived Outcome Risk Assessment Score (PANDORA): Development of a Simple Predictive Risk Score for 30-Day In-Hospital Mortality Based on Demographics, Clinical Observation, and Nutrition
Source: PLoS One. 2015 May 22;10(5):e0127316. doi: 10.1371/journal.pone.0127316 (PMC4441510; doi:10.1371/journal.pone.0127316)
Supplement: S1 Table — (DOCX) [file pone.0127316.s002.docx]

| Country | Ethical committee |
| --- | --- |
| Austria | Medical University Vienna |
| Belgium | Erasme Hospital Brussels |
| Belgium | Comité d’Ethique Hospitalo-Facultaire Universitaire de Liège |
| Brasil | Universidade Catolica de Pelotas UCPEL |
| Canada | Research Ethics Board of North York General Hospital Toronto |
|  | Fraser Health Research Ethics Board |
|  | University of Manitoba Research Ethics Board |
| Chile | Local EC not required |
| China | Nanjing General Hospital of Nanjing Military Command |
| Colombia | Fundación Cardiovascular de Colombia |
|  | Clinica San Fransisco de Asis |
|  | Hospital San Jose |
|  | Oncologos del Occidente |
|  | Clinica La estancia |
|  | Clinica del Occidente |
|  | Hospital Simon Bolivar |
|  | Hospital Departamental de Villavicencio |
|  | Clinica Cooperativa |
|  | Amyser Hogar Gerontologico |
|  | Hospital Medico Quirurgico |
|  | Clinica Juan N Corpas |
|  | Hospital Erasmo Meoz |
|  | Clinica Universitaria Colombia |
|  | Hospital Universitario San Rafael |
| Croatia | Ethic Committee is University Hospital Centre Zagreb Ethic Committee. |
| Czech republic | local EC approval not required |
| Estonia | Ethics Review Committee on Human Research of the University of Tartu, Estonia |
| Finland | EC in Espoo: Town Espoo, Social and Health Services, Development Unit |
|  | The Pirkanmaa Hospital District’s Science Centre |
|  | Kuopio University Hospital's Science Service Center |
| France | Comité de Protection des Personnes Sud Mediterranee, Nice |
| Germany | Ethik Kommission Charité Universitätsmedizin Berlin |
|  | Ethik Kommission der Albert-Ludwigs Universität, Freiburg |
| Greece | Ethical Review Board of “Laiko” General Hospital of Athens |
|  | Korgialenio Benakio General Hospital |
|  | Evaggelismos Hospital |
|  | General Hospital of Kavala |
|  | General hospital of Thessaloniki |
|  | General Hospital-Eftychios Patsidis |
|  | GH Asklepieio Voulas |
|  | Hippokration Hospital of Athens |
|  | Konstantopouleio general hospital of Nea Ionia |
|  | Korgialenio Benakio General Hospital |
|  | Papageorgiou General Hospital |
|  | Saint Savvas Cancer Hospital, Athens |
|  | Sotiria General Hospital |
|  | University Hospital of Heraklion |
|  | University Hospital of Larissa ,Thessaly |
|  | Volos General Hospital |
| Israel | Rabin Medical Center |
| Italy | Comitato Etico Presidio Ospedaliero Pietra Ligure - Albenga (Savona) |
| Japan | Teikyo University School of Medicine |
|  | Ethics Committee of Obase Hospital, Fukuoka Japan |
|  | Ethics Committee Toho University Omori Medical Center |
|  | Ethics Committee Teikyo University School of Medicine |
|  | Ethics Committee Toho University Omori Medical Center, Tokyo |
|  | Ethics Committee of Cancer Institute Hospital, Tokyo Japan |
|  | Ethics Committee  Niigata University School of Medicine, Niigata Japan |
|  | Ethics Committee Chita　City Hospital, Aichi Japan |
|  | Research Ethics Committee of Kawasaki Medical School and Hospital |
|  | Ethics Committee Takasaki General Medical Center, Gunma, Japan |
|  | Institutional Review Board of Haramachi Red-Cross Hospital, Gunma Japan |
|  | The Ethical Committee of Kurume University |
|  | Ethics Committee  Nagano Red Cross Hospital,　Nagano Japan |
|  | Ethics Committee of Iwate Medical University, School of Medicine |
|  | Fujita Health University Ethical Review Board for Epidemiological and Clinical Studies |
|  | Research Ethics Committee, Kinki Central Hospital, Hyogo, Japan |
| Latvia | Ethics Committee for Clinical Research at Pauls Stradins Clinical University Hospital Development Society |
|  | Medical and biomedical research Ethics Committee of the Riga East Clinical University hospitals Support Foundation |
|  | Ethics Committee for Clinical Research at Pauls Stradins Clinical University Hospital Development Society |
| Lithuania | Bioethical commitee, Vilnius University Hospital" Santariskiu Clinics" |
| Mexiko | Comite Hospitalario de Êtica e Investigaciòn, del Centenario Hospital Miguel Hidalgo |
| Netherlands, the | local EC approval not required |
| Norway | Data Protection Officer, Norwegian Health Act, North-Norway University Hospital |
| Poland | not needed |
| Portugal | local EC approval not required |
| Romania | Information of local ethical committee |
| Slovenia | University Medical Center Maribor, Slovenia |
| Spain | Comité Etico de investigacion clinica (CEIC) Hospital clinic - Provincial de Barcelona |
|  | Comité Etico de investigacion clinica (CEIC) Hospital Universitario La Paz - Madrid |
|  | COMITÉ ÉTICO DE INVESTIGACIÓN CLÍNICA  HOSPITAL CLÍNICO SAN CARLOS |
|  | CEIC Consorci Sanitari de Terrassa |
|  | The Clinical Research Ethics Committee of the Hospital Universitari de Bellvitge |
| Switzerland | Commission d'Ethique, HUG |
|  | Commission D’Ethique de la recherche clinique, Lausanne |
| Turkey | Ankara Training and Research Hospital Institutional Review Board |
|  | University of Uludag Faculty of Medicine Ethics Committee |
| United States | Veterans Administration Memphis |
|  | Advocate Healthcare system |
