# Supplementary material for: The Patient- And Nutrition-Derived Outcome Risk Assessment Score (PANDORA): Development of a Simple Predictive Risk Score for 30-Day In-Hospital Mortality Based on Demographics, Clinical Observation, and Nutrition
Source: PLoS One. 2015 May 22;10(5):e0127316. doi: 10.1371/journal.pone.0127316 (PMC4441510; doi:10.1371/journal.pone.0127316)
Supplement: S2 Table — Reference categories are marked in bold characters. (DOCX) [file pone.0127316.s003.docx]

| Variable(s) | Classes |
| --- | --- |
| Sex | **Female**; male |
| Age group | **<40**; 40-50; 50-60; 60-70; 70-80; 80-90; >=90 |
| BMI group | <18.5; 18.5-25; 25-30; 30-35; **35-40**; >=40 |
| Weight 5 years ago | weight in kg |
| Decrease of weight within the last three months | **<5 kg**; 5-10 kg; >=10 kg |
| Main patient group admitted | geriatric; **surgical**; neurological; internal; others |
| Duration since hospital admission | **<=14 days**; >14 days |
| Any stay in Intensive Care Unit | yes; **no** |
| Waiting for operation | yes; **no** |
| Can you walk alone? | **yes**; no-only with assistance; no-I stay in bed |
| Did anyone help filling questionnaires? | yes; **no** |
| Affected organs according to ICD10 categories:  brain/nerves; eye/ear; nose/throat; heart/circulation; lung; liver; gastrointestinal tract; kidney/urinary tract/female genital tract; endocrine system; skeleton/bone/muscle; blood/bone marrow; skin; ischemia; cancer; infection; other; | EACH yes; **no** |
| Comorbidities:  none; diabetes I/II; stroke; COPD; myocardial infarction; cardiac insufficiency; other; | EACH yes; **no** |
| Drugs | yes; **no** |
| Fluid status | Overloaded; **normal**; dry |
| What did you eat last week? | **normal**; bit less; less than half of normal; less than a quarter of normal |
| Reasons for eating less last week:  none; loss of appetite; nausea; problems with swallowing/chewing; other; | EACH yes; **no** |
| What did you eat at lunch or dinner today? | **all**; 1/2; 1/4; nothing/allowed to eat; nothing/not allowed to eat |
| Reasons for meal not eaten:  none; not hungry; nausea/vomiting; can’t without help; tired; normally eat less; didn’t like smell; didn’t like taste; | EACH yes; **no** |
| Additional nutrition | yes; **no** |
