# Supplementary material for: The Patient- And Nutrition-Derived Outcome Risk Assessment Score (PANDORA): Development of a Simple Predictive Risk Score for 30-Day In-Hospital Mortality Based on Demographics, Clinical Observation, and Nutrition
Source: PLoS One. 2015 May 22;10(5):e0127316. doi: 10.1371/journal.pone.0127316 (PMC4441510; doi:10.1371/journal.pone.0127316)
Supplement: S3 Table — The relationship between the extended score and in-hospital mortality within 30 days after the day of the cross-sectional survey is given by the equation: logit = -6.79 + 0.1091 x extended score. The probability of death is given by the equation: Probability of death = elogit / (1+elogit). (DOCX) [file pone.0127316.s004.docx]

| **Variable** | **Groups** | **Score** |
| --- | --- | --- |
| Sex | male | 2 |
| Age | <40 | 0 |
|  | 40-50 | 5 |
|  | 50-60 | 7 |
|  | 60-70 | 8 |
|  | 70-80 | 10 |
|  | 80-90 | 14 |
|  | >=90 | 17 |
| Body Mass Index (BMI) | <18.5 | 6 |
|  | 18.5-25 | 4 |
|  | 25-30 | 2 |
|  | 30-35 | 0 |
|  | 35-40 | 0 |
|  | >40 | 3 |
| Can you walk? | Walk without assistance | 0 |
|  | Only with assistance | 6 |
|  | I stay in bed | 11 |
| Eating last week? | Normal | 0 |
|  | Bit less | 0 |
|  | Less than half | 2 |
|  | Less than a quarter | 3 |
| What did you eat today? | All | 0 |
|  | Half | 3 |
|  | Quarter | 7 |
|  | Nothing, Allowed | 10 |
|  | Nothing, Not allowed | 6 |
| Receiving additional nutrition | Yes | 4 |
| Days since hospital admission | >=14 | 3 |
| Main patient group admitted | Internal | 6 |
|  | Surgery | 0 |
|  | Geriatrics | 4 |
|  | Neurology | 3 |
|  | Others | 5 |
| Diseased Organ -> Multiple answers possible | Lung | 3 |
|  | Liver | 4 |
|  | Gastrointestinal tract | -2 |
|  | Skeleton/bone/muscle | -3 |
|  | Cancer | 8 |
|  | Other | 0 |
| Fluid status | Dehydrated | 6 |
|  | Normal | 0 |
|  | Overload | 9 |
| **extended score** | **sum** |  |
